# Synergetic role of TRPV4 inhibitor and mechanical loading on reducing inflammation

**DOI:** 10.3389/fimmu.2024.1456042

**Published:** 2025-01-07

**Authors:** Parto Babaniamansour, Diego Jacho, Agustin Rabino, Rafael Garcia-Mata, Eda Yildirim-Ayan

**Affiliations:** ^1^ Department of Bioengineering, College of Engineering, University of Toledo, Toledo, OH, United States; ^2^ Department of Biological Sciences, University of Toledo, Toledo, OH, United States

**Keywords:** transient receptor potential vanilloid 4 (TRPV4), inhibition, mechanical loading, macrophage polarization, pro-inflammatory (M1) macrophages, anti-inflammatory phenotype, mitogen-activated protein kinase (MAPK), three-dimensional (3D) collagen matrix

## Abstract

Resolution of inflammation is essential for normal tissue healing and regeneration, with macrophages playing a key role in regulating this process through phenotypic changes from a pro-inflammatory to an anti-inflammatory state. Pharmacological and mechanical (mechanotherapy) techniques can be employed to polarize macrophages toward an anti-inflammatory phenotype, thereby diminishing inflammation. One clinically relevant pharmacological approach is the inhibition of Transient Receptor Potential Vanilloid 4 (TRPV4). This study investigates the effects of various mechanical loading amplitudes (0%, 3%, and 6%) and TRPV4 inhibition (10 µM RN-1734) on the phenotypic commitments of pro-inflammatory (M1) macrophages within three-dimensional (3D) collagen matrices. M1 macrophages exposed to 3% mechanical strain exhibited upregulated pro-inflammatory responses, including increased pro-inflammatory gene expression and enhanced proteolytic activity within the extracellular matrix. TRPV4 inhibition partially mitigated this inflammation. Notably, 6% mechanical strain combined with TRPV4 inhibition suppressed Mitogen-Activated Protein Kinase (MAPK) expression, leading to reduced pro-inflammatory gene expression and increased anti-inflammatory markers such as CD206. Gene expression analysis further demonstrated significant reductions in pro-inflammatory gene expression and a synergistic promotion of anti-inflammatory phenotypes under TRPV4 inhibition at 6% mechanical strain. Surface protein analysis via immunohistochemistry confirmed these phenotypic shifts, highlighting changes in the expression of CD80 (pro-inflammatory) and CD206 (anti-inflammatory) markers, alongside F-actin and nuclear staining. This research suggests that TRPV4 inhibition, combined with specific mechanical loading (6%), can drive macrophages toward an anti-inflammatory state, thereby may promote inflammation resolution and tissue repair.

## 1 Introduction

Macrophages are highly dynamic cells involved in host defense, maintenance of tissue homeostasis, and resolution of inflammation (1, 2). Therapies to resolve inflammation and promote tissue regeneration often target macrophages to block pro-inflammatory (M1) activation pathways using various stimuli and to modulate their differentiation towards an anti-inflammatory (M2) phenotype (3).

Macrophages are also highly mechanosensitive, meaning they can sense and respond to mechanical stimuli within their microenvironment. Mechanical stimuli, including stretch, compression, and interstitial flow, influence macrophage polarization, driving shifts between pro-inflammatory and anti-inflammatory phenotypes (2, 4). These phenotypic transitions under mechanical loading are critical for inflammation resolution, tissue healing, and regeneration particularly for the tissues exposed to constant mechanical loading such as musculoskeletal tissues. Several seminal studies including ours (5) highlighted the role of mechanical stimuli in macrophage polarization. For instance, Tu et al. (6) seeded murine macrophages on flexible membrane and applied mechanical strain up to 15% for 8 hours. They demonstrated that 15% mechanical strain increased NF-κB activation and pro-inflammatory cytokine secretion. The studies conducted by Wang et al. (7) demonstrated that mechanical pressure applied on murine macrophages within the plates could initially promote a pro-inflammatory phenotype, then shift to an anti-inflammatory of state over time. These findings underscore the critical role of mechanical loading in macrophage mechanotransduction and polarization.

Mechanotransduction in macrophages involves a complex interplay of mechanosensors such as adhesion molecules, cytoskeletal components, and ion channels, including the Transient Receptor Potential Vanilloid 4 (TRPV4) channel. TRPV4 is a mechanosensitive ion channel activated by mechanical stimuli, temperature, chemical agents, and endogenous or exogenous ligands (8, 9). Activation of TRPV4 induces conformational changes that lead to Ca²^+^ influx, which is crucial in regulating macrophage behavior and inflammation (10). Pharmacological inhibition of TRPV4 has been shown to attenuate pro-inflammatory responses (11–13). Dalsgaard et al. (14) reported that TRPV4 antagonists could prevent sepsis-induced increases in pro-inflammatory cytokines in mice, highlighting its potential as a pharmacological intervention in inflammatory diseases. Yet, the concurrent effects of TRPV4 inhibition and mechanical loading on macrophage polarization within 3D environments have not been investigated. Understanding these combined effects could unlock new strategies for regulating inflammation and promoting tissue repair.

Towards this end, the current study aims to explore the synergistic effects of mechanical loading and TRPV4 inhibition on pro-inflammatory macrophages encapsulated within 3D collagen matrices. By applying varying mechanical strains (0%, 3%, and 6%) and using TRPV4 inhibitors, we assess the macrophage phenotypic shifts and associated mechanotransduction pathways at both the gene and protein levels. Techniques such as ELISA, RT-PCR, and immunofluorescence staining of surface markers (CD80 and CD206) are employed to evaluate the macrophage response. Structural assessments, including Masson’s trichrome staining and collagen hybridizing peptide (CHP) staining for degraded collagen, further elucidate the impact of these interventions on the macrophage-matrix interactions. This comprehensive approach aims to reveal how TRPV4 inhibition and mechanical loading can modulate macrophage behavior, offering potential therapeutic insights for inflammation resolution and tissue regeneration.

## 2 Materials and methods

### 2.1 Human macrophage cultivation and pharmacological inhibition of TRPV4 ion channel

The human pro-monocytic cell line U937 (ATCC, Manassas, VA, USA) was cultured with a complete RPMI 1640 medium (ATCC, Manassas, VA, USA) containing 10% heat-inactivated fetal bovine serum (FBS; ThermoFisher, Waltham, MA, USA), 2 mM L-glutamine, 10 mM HEPES, 1 mM sodium pyruvate, 4.5 g/L glucose, and 1.5 g/L sodium bicarbonate at 37°C, 5% CO_2_. The media was changed every 2-3 days. Upon reaching confluency, U937 monocytes were incubated with 100 ng/mL phorbol 12-myristate 13-acetate (PMA) (Sigma-Aldrich, St. Louis, MO, USA) for 24 hours for differentiation into naïve macrophages (M0). Subsequently, M0 macrophages were differentiated into a pro-inflammatory (M1) phenotype through culturing them in media supplemented with 20 ng/mL interferon-gamma (IFNγ, Peprotech, Cranbury, NJ, USA) and 100 ng/mL lipopolysaccharide (LPS) (Sigma, Ronkonkoma, NY, USA) for 24 hours based on our established protocol (15).

A selective TRPV4 ion channel inhibitor, 10 µM RN-1734 (Millipore Sigma, R0658) was used as a TRPV4 antagonist based on its selectivity and sensitivity (16). In addition, our prior study demonstrated that10 µM RN-1734 concentration was enough to block the TRPV4 channel without negatively affecting the cell viability (16). The RN-1734 was added into the complete cell culture media one day before the cellular encapsulation within 3D matrix. In terms of TRPV4 ion channel treatment, two groups were assigned, namely TRPV4(-) and TRPV4(0). In TRPV4(-) group, cells were treated with TRPV4 inhibitor prior to encapsulation. In TRPV4 (0), cells were cultured in a complete media without TRPV4 inhibitor and served as a control group. For both groups, upon encapsulating the cells within the 3D matrix, the mechanical loading was applied with various mechanical strain amplitudes (0,3, and 6%).

### 2.2 Macrophage-laden 3D matrix synthesis and mechanical loading

**Figure 1:**
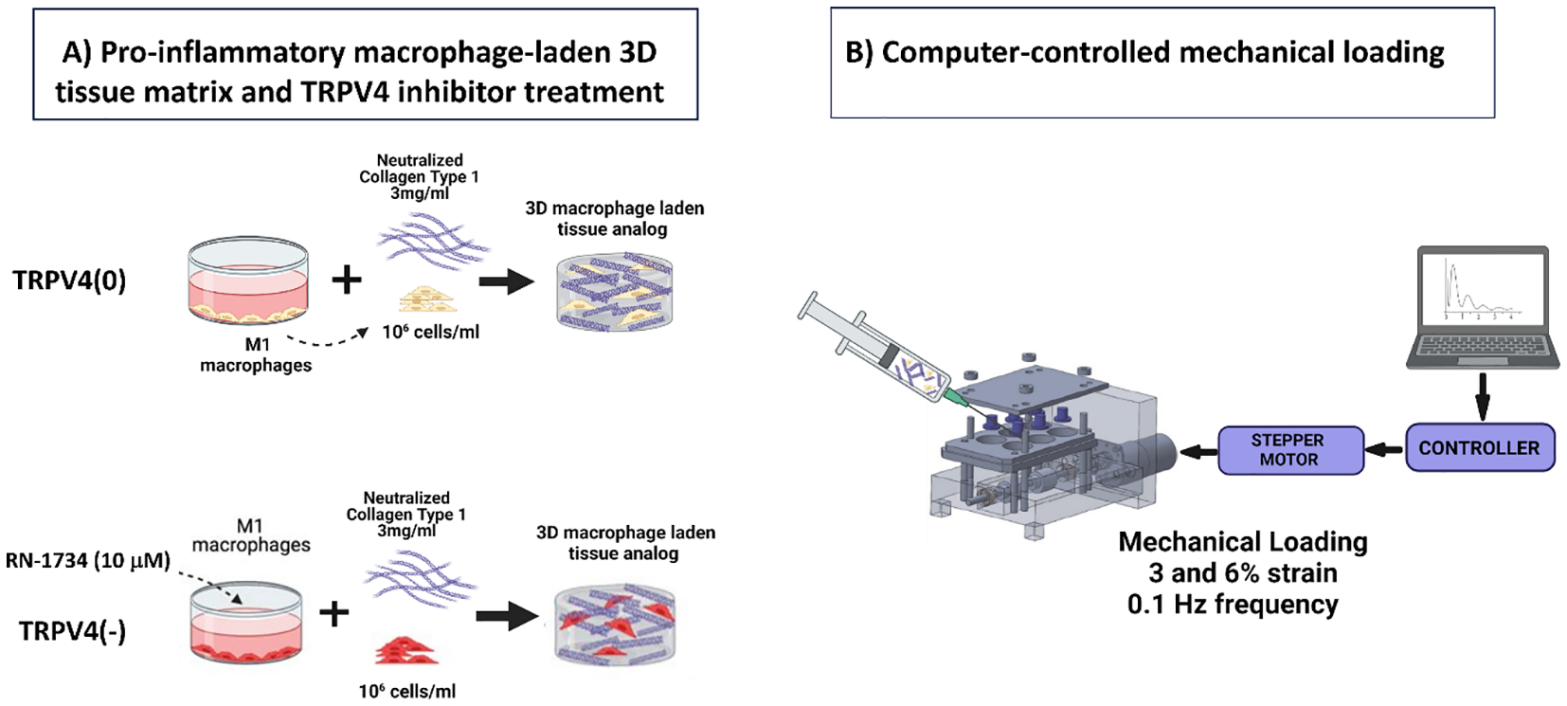
**(A)** Schematic illustrations detailing the fabrication process of a macrophage-laden 3D collagen matrix for TRPV4(0) and TRPV4(-) groups. **(B)** The schematic representation of the systems involved in computer-controlled mechanical loading to the macrophage-laden collagen matrix.

To synthesize macrophage-laden 3D collagen matrix, the M1 macrophages with and without TRPV4 inhibitor treatment were encapsulated within neutralized 3 mg/mL collagen type-I (Ibidi, WI, USA) at a seeding density of 1x10^6^ cells/mL, following our established protocols (16, 17). Collagen type I was chosen as the matrix material, given its prevalence in the extracellular matrix (ECM) of most tissues (18). Some of the M1 macrophage-loaded 3D matrices were placed in mechanical loading chambers to apply 3% and 6% strains, while others were placed in the chambers without any mechanical strain (0%). All samples were then placed in an incubator (37C and 5%CO2) for 24 hours for collagen polymerization and cellular acclamation. **Figure 1A** demonstrates the major steps followed for creating macrophage-laden 3D tissue matrices.

Following 24-hour incubation, the various mechanical strains were applied to macrophage-laden 3D collagen matrix using a custom-built and confirmed, computer-controlled mechanical loading platform called EQUicycler (**Figure 1B**). The EQUicycler, a specialized mechanical loading platform, is able to apply pre-defined mechanical strain amplitudes (up to 15%) and frequencies (up to 2Hz) to the cell-laden matrices, 3D tissues, and ex-vivo organs including human intervertebral discs, porcine (IVDs) and rat or mouse hindlimbs (17–20). In this study, 3% or 6% mechanical strain amplitudes with 0.1 Hz frequency were applied for 2 hours each day for 4 days to the macrophage-laden 3D collagen matrix. The strain amplitudes were chosen based on the physiological strain experienced by most of the musculoskeletal tissues within the human body (15).

To understand the combined role of mechanical loading and TRPV4 inhibitor and to decipher their solo effect on macrophage polarization, six groups of samples were used. The first group was the control group called TRPV4(0)-0% which neither TRPV4 inhibitor nor mechanical strain were applied. The other groups are TRPV4(0)-3% and TRPV4(0)-6% which were mechanically strained with 3% and 6% amplitudes but no TRPV4 inhibitor applied. The TRPV4 inhibitor groups are without and with mechanical loading, namely TRPV4(-)-0%, TRPV4(-)-3% and TRVP4(-)-6%.

### 2.3 Structural changes in macrophage-laden tissue matrix upon combined mechanical loading and TRPV4 inhibition

The structural changes within the macrophage-laden 3D collagen tissue matrix were evaluated by assessing the collagen fibers and degraded proteolyzed collagen matrix using Masson’s trichrome staining and B-Collagen hybridizing peptide (B-CHP) analysis, respectively. Briefly, on characterization day, the samples were harvested and fixed with 10% formalin (HT501128-4L, SIGMA) overnight followed by a serious dehydration process with graded ethanol (ThermoFisher) and embedded in paraffin for sectioning. The next day, the samples were sectioned to a thickness of 5μm using a microtome (GMI-Reichert Jung 820 II) and affixed to a glass microscopic slide (Mercedes Medical MER 7200/45/BL). For assessing collagen fibers, the histological slides were stained with Masson’s trichrome staining then washed with PBS and visualized using a digital microscope (Keyence).

For observing the possible collagen degradation, 8 μm thick histological slides from all experimental groups and control groups were mounted on microscopic slides and subjected to an overnight incubation at 55°C. The manufacturer’s protocols were followed to tag the degraded collagen (50 μM; B-CHP #BIO-300; 3Helix, USA) (21). Briefly, the samples underwent an overnight incubation with B-CHP (20 μM) at 4°C and were then washed with PBS for 5 minutes at room temperature. Then, the slides were further incubated in a PBS solution containing 1% BSA with 0.005 mg/mL of Alexa Fluor™ 488 streptavidin conjugate (Thermo Fisher, A12379) for 2 hours at room temperature. After a 5-minute PBS wash, imaging was conducted using the OLYMPUS IX71 microscope and the cellSens Dimension software. Subsequent post-processing and analysis were performed using ImageJ software. Specifically, fluorescent images from four fields of view per treatment group were processed to measure fluorescent integrated density per area. The integrated density values were adjusted by subtracting background integrated density values and dividing them by the area value to determine the CHP fluorescent intensity per unit area (CHP signal) for each treatment group.

### 2.4 Mitogen-activated protein kinase activity changes in macrophages within 3d collagen matrix upon combined mechanical loading and TRPV4 inhibition

The activation of crucial pathways involved in immunomodulation, such as the mitogen-activated protein kinase (MAPK) pathway and its key components, including the extracellular signal-regulated kinase 1 and 2 (ERK1/2) and p38 MAPKs were assessed using enzyme-linked immunosorbent assays (ELISA).

To understand the changes in p38-MAPK in each strain group, p38 MAPK (Total) ELISA Kit (KHO0061, ThermoFisher) was used. Briefly, the macrophage-laden 3D matrices were lysed using a RIPA buffer (R0278-50ML, Sigma-Aldrich) and followed by mechanical agitation using a homogenizer for complete lysis. Each sample was centrifuged at 1000 RPM for 10 minutes at 4°C and then the supernatant was transferred to a new tube and the protein concentration was measured with BCA protocol using Pierce™ Bovine Serum Albumin Standard Ampules (23209, ThermoFisher). For each strain group, the supernatant volume was diluted in Standard Diluent Buffer to ensure uniform protein concentration across all groups. 100 ml of pre-diluted samples, control, and standards were added to the antibody-coated well plate incubated for 2 hours at RT and washed 4 times with the wash buffer. Then, 100 ml of p38 MAPK (Total) Detection Antibody solution was added to each well and incubated for another hour at RT and washed 4 times with the washing buffer. Then, 100 ml of 1X Anti-Rabbit IgG HRP Solution was added to each well to detect bounded antibodies and provide more specificity. After 30 minutes of the incubation at RT, the plate was washed for 4 times with wash buffer and 100 ml of stabilized chromogen was added to each well for better visualization and the plate was incubated for 30 minutes at RT. Finally, after adding 100 ml of stop solution to the well plate and incubating it for 2 hours at RT, the absorbance was read at 450 nm and the p38 MAPK concentration was calculated based on the standard curve.

To find the expression of ERK1/ERK2 in each strain group, ERK1/ERK2 (Total/Phospho) Multispecies InstantOne™ ELISA Kit (85-86013-11, ThermoFisher) was used. The cell lysates were prepared using Cell Lysis Mix (1X, 85-86013-11, ThermoFisher) and subsequently shaken at 300 RPM for 10 minutes. Then,10 ml of sample lysate was added to the well plate followed by 10 ml of antibody cocktail including Capture Antibody Reagent and Detection Antibody Reagent. The plate was incubated for one hour at room temperature on a microplate shaker set to 300 RPM. The wells were washed three times with wash buffer, then incubated with 10 µL of Detection solution for one hour on a microplate shaker (300 RPM). Then, 20 µL of Stop Solution was added to each well, and the plate was read at an absorbance of 450 nm.

### 2.5 Phenotypic changes in macrophages within 3D tissue matrix upon combined mechanical loading and TRPV4 inhibition

To study the effect of TRPV4 inhibitor and/or mechanical loading on phenotypic changes in macrophages, gene expression analysis using quantitative real-time polymerase chain reaction (qRT-PCR) and cell surface marker analysis using immunostaining were performed.

#### 2.5.1 Gene expression analysis

The RNA extraction was conducted using TRIzol reagent (ThermoFisher Scientific, USA), followed by reverse transcription with the Omniscript RT kit (Qiagen, Germantown, MD, USA) as per the manufacturer’s instructions. The qRT-PCR was conducted using the SYBR Green PCR master mix (ThermoFisher Scientific, USA) to detect the expression of pro- and anti-inflammatory genes. The relative fold changes between the experimental groups and control groups were obtained using the ΔΔCt method with glyceraldehyde-3-phosphate dehydrogenase (GAPDH) serving as the housekeeping gene for normalization. The qRT-PCR experiments were conducted using the iCycler iQ detection system (Bio-Rad, USA) with a total of 35 thermocycling cycles. Primer sequences were obtained from published literature, as listed in **Table 1**, and Integrated DNA Technologies (IDT, USA). The relative fold changes of pro- and anti-inflammatory genes further were subjected to additional analysis and visualization utilizing open-source software (http://www.heatmapper.ca). The heatmaps were generated employing the average linking method with the Euclidean method to compare the gene expressions of macrophages within the 3D tissue matrix upon TRPV4 inhibitor and/or mechanical loading and control groups.

**Table 1:** Forward and reverse primers for quantitative real-time polymerase chain reaction.

Gene	Forward Primer	Reverse Primer	Ref
*COX2*	5′-CGGTGTTGAGCAGTTTTCTCC-3′	5′-AAGTGCGATTGTACCCGGAC-3′	(22)
*MMP13*	5'-ACTGAGAGGCTCCGAGAAATG-3'	5'-GAACCCCGCATCTTGGCTT-3'	(23)
*TNF-α*	5'-AGAGGGAAGAGTTCCCCAGGGAC-3'	5'-TGAGTCGGTCACCCTTCTCCAG-3'	(22)
*IL-1β*	5'-CCAGCTACGAATCTCGGACCACC-3'	5'TTAGGAAGACACAAATTGCATGGTGAAGTCAGT-3'	(24)
*CD163*	5'-TCTGTTGGCCATTTTCGTCG-3'	5'TGGTGGACTAAGTTCTCTCCTCTTGA-3'	(22)
*CCL18*	5'-AAGAGCTCTGCTGCCTCGTCTA-3'	5'-CCCTCAGGCATTCAGCTTAC-3'	(25)
*GAPDH*	5’-AGAAGGCTGGGGCTCATTTG-3’	5’-AGGGGCCATCCACAGTCTTC-3’	(22)

#### 2.5.2 Cell surface markers analysis (immunostaining)

The macrophage-laden 3D tissue matrices in all experimental groups and control groups underwent fixation in 10% formalin, dehydration in graded ethanol, and embedding in paraffin. Then, the tissue matrices were sliced with a thickness of 40 μm using a microtome (GMI-Reichert Jung 820 II), mounted on a glass microscopic slide (Mercedes Medical MER 7200/45/BL) and kept in the oven overnight at 80°C. The following day, slides were treated to remove residual paraffin by immersion in xylene for 2 hours. Subsequently, they underwent a series of ethanol washes (100%, 95%, 70%, and 50%) followed by rinsing in diH2O for post-deparaffinization and rehydration. The slides were then subjected to antigen retrieval by incubating them in a solution of trypsin and calcium chloride (0.1% (v/v) trypsin from ATCC, USA, and 0.1% (w/v) calcium chloride from Sigma-Aldrich, USA) at 37°C for 30 minutes. Heat-mediated antigen retrieval was performed by immersing the slides in citrate buffer (10 mM sodium citrate in 0.05% Tween 20) at 95°C for 30 minutes. Following permeabilization with Triton X-100 for 10 minutes, samples were washed in PBS and then blocked in a solution containing 2.5% (v/v) goat serum (Invitrogen) and 0.2% Tween for 20 minutes to prevent non-specific antibody binding.

Overnight incubation at 4°C was conducted with mouse monoclonal CD80 antibody (1:100, Invitrogen, TA501575) and recombinant rabbit CD206 antibody (1:100, ThermoFisher, MA1-35936), selected as pro-inflammatory and anti-inflammatory macrophage-related surface markers, respectively. Following washing in PBS and 0.2% Tween solution, slides were re-blocked in the goat serum solution for 20 minutes at room temperature. Subsequently, slides were incubated with Alexa Fluor 488 Phalloidin (1:200, ThermoFisher, A12379), goat anti-rabbit IgG (H+L) Highly Cross-Adsorbed Secondary Antibody Alexa Fluor 594 (1:200, ThermoFisher, A32740), and goat anti-mouse IgG (H+L) Highly Cross-Adsorbed Secondary Antibody, Alexa Fluor Plus 800 (1:200, ThermoFisher, A32730) for 4 hours and 30 minutes. Finally, slides were treated with DAPI (4’,6-Diamidino-2-Phenylindole) dye (1:1000, Life Technologies) in PBS for 30 minutes and washed with 0.2% Tween.

#### 2.5.3 Anti-TRPV4 antibody staining

After tissue sectioning and slide preparation, the slides were incubated overnight at 4°C with anti-TRPV4 antibody (1:80) (EMD Millipore, MABS466) to visualize the expression of TRPV4 in control and experimental groups. The next day, a PBS and 0.2% Tween solution wash was performed, followed by blocking in 0.4% BSA and 0.2% Tween solution for 20 minutes at room temperature (RT). After blocking, slides were incubated for 2 hours at RT with Alexa Fluor Plus 800 (ThermoFisher, A32730) at a dilution of 1:50, followed by washing with 0.2% Tween solution.

#### 2.5.4 Confocal imaging

Imaging was conducted using a 63x oil or 20x dry objective with a Leica Stellaris 5 confocal system equipped with HyD detectors and the LASX software. Confocal images from at least three independent studies were processed to visualize each cell’s nuclei, filamentous actin (F-actin), and CD80, CD206, and TRPV4 channel expressions. The anti-TRPV4 antibody confocal images were then post-processed and analyzed with ImageJ software to measure a fluorescent signal associated with the secondary antibody. The integrated density values obtained for each cell were corrected by subtracting the background signal and normalized by dividing by cell surface area.

### 2.6 Statistical analysis

At least three independent samples were used for this study (n=3). Six technical replicates were employed for all assays, and statistical analysis was performed using RStudio. Statistical significance was assessed through one-way analysis of variance (ANOVA) and *post-hoc* analysis (Tukey test) or Student’s t-test when applicable. In the graphs, error bars demonstrate standard error unless otherwise indicated.

## 3 Results

### 3.1 TRPV4 channel expression upon TRPV4 inhibition and mechanical loading

**Figure 2:**
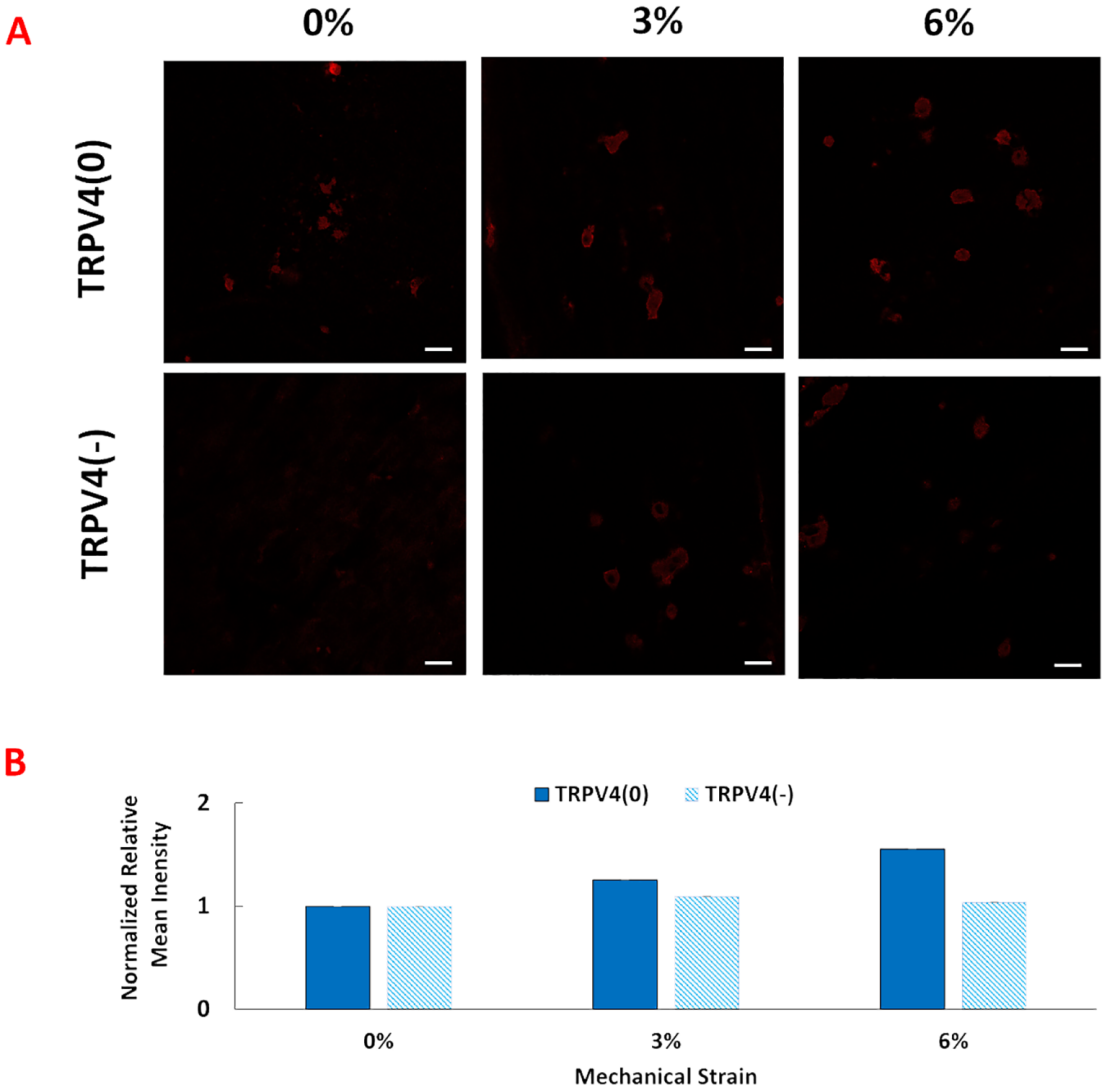
**(A)** Anti-TRPV4 antibody staining of the pro-inflammatory macrophages encapsulated within the 3D tissue matrix upon 0%, 3%, and 6% mechanical strain and TRPV4 inhibition. The scale bar is 20 mm. **(B)** Quantification of anti-TRPV4 antibody staining from image analysis. Relative fluorescence intensity was normalized with the fluorescence intensity of control group, TRPV(0)-0% group.

To investigate the changes in TRPV4 expression upon TRPV4 inhibition and/or mechanical loading at 0%, 3%, and 6% strains, TRPV4 ion channels were tagged with an anti-TRPV4 antibody. **Figure 2** shows the immunofluorescence images of TRPV4 channels with (TRPV4(-)), and without TRPV4 inhibitor (TRPV4(0)), upon various mechanical loading amplitudes.

The anti-TRPV4 antibody staining images and their quantification demonstrated that TRPV4 expression without presence of inhibitor increased progressively with mechanical strains from 0% to 6%. However, in the presence of a TRPV4 inhibitor (TRPV4(-)), TRPV4 expression was reduced for 3% and 6% mechanical loading groups with greater reduction in TRPV4(-)-6% mechanical loading group., even under 3% and 6% mechanical loading conditions. Write down the quantification.

### 3.2 Structural changes in M1-laden 3D collagen matrix upon TRPV4 inhibition and mechanical loading

The structural changes within the collagen fiber density and degradation within the collagen upon.

TRPV4 inhibition and mechanical loading were visualized for macrophage-laden 3D collagen matrices subjected to 0%, 3%, and 6% mechanical strain, both with and without TRPV4 inhibition.

**Figure 3:**
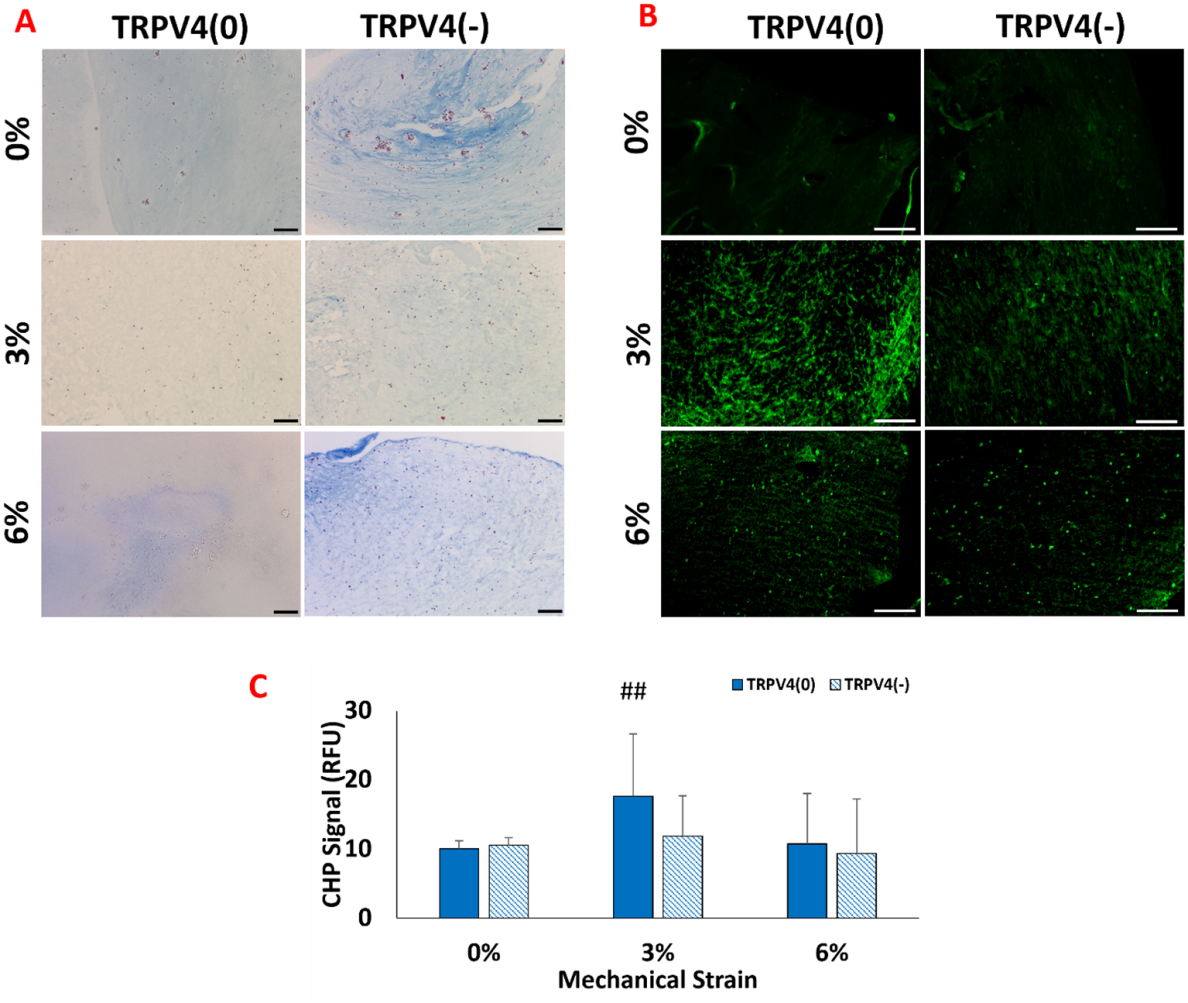
**(A)** Histological images of collagen fibers (blue) and cell nuclei (purple) staining with the Masson’s trichrome **(B)** Fluorescence images of degraded collagen fibers within the 3D matrix stained with B-CH. The scale bar represents 200 µm. **(C)** Quantification of degraded collagen from image analysis. ## shows that there is a statistical difference has a significance level of p<0.005 between control (TRPV4(0)-0%), n=5.

The changes in collagen density upon mechanical loading and TRPV4 inhibitor treatments were visualized by Masson’s trichrome staining (**Figure 3A**), while the collagen degradation was visualized and quantified with B-CHP staining (**Figures 3B, C**).

**Figure 3A** shows that the M1 macrophages exposed to 3% mechanical strain without TRPV4 inhibitor, TRPV4(0)-3% group, demonstrated a loose collagen fiber structure compared to both the control group (TRPV4(0)-0%) and all other experimental groups. The low collagen content observed in the TRPV4(0)-3% group is partially compensated by the TRPV4 inhibitor, as seen in the TRPV4(-)-3% group. Overall, the collagen fibers encapsulating M1 macrophages treated with the TRPV4 inhibitor (TRPV4(-) group) were thicker compared to the TRPV4(0) group. This indicates that blocking the TRPV4 channel in M1 macrophages enhances collagen density perhaps through preventing collagen degradation and by modulating macrophage phenotype. The images and image analysis of degraded collagen matrices (**Figures 3B, C**) revealed that the highest level of degradation occurred in the 3% mechanical strain group without the TRPV4 inhibitor (TRPV4(0)-3%), consistent with the observations from Masson’s trichrome staining. While there was slight difference in degraded collagen between the 0% groups with or without the TRPV4 inhibitor, the addition of the TRPV4 inhibitor in both the 3% and 6% mechanically loaded matrices led to a reduction in the degree of collagen denaturation and degradation (**Figure 3B**).

### 3.3 Combined TRPV4 inhibition and mechanical strain modulate macrophage MAPK signaling pathway

**Figure 4:**
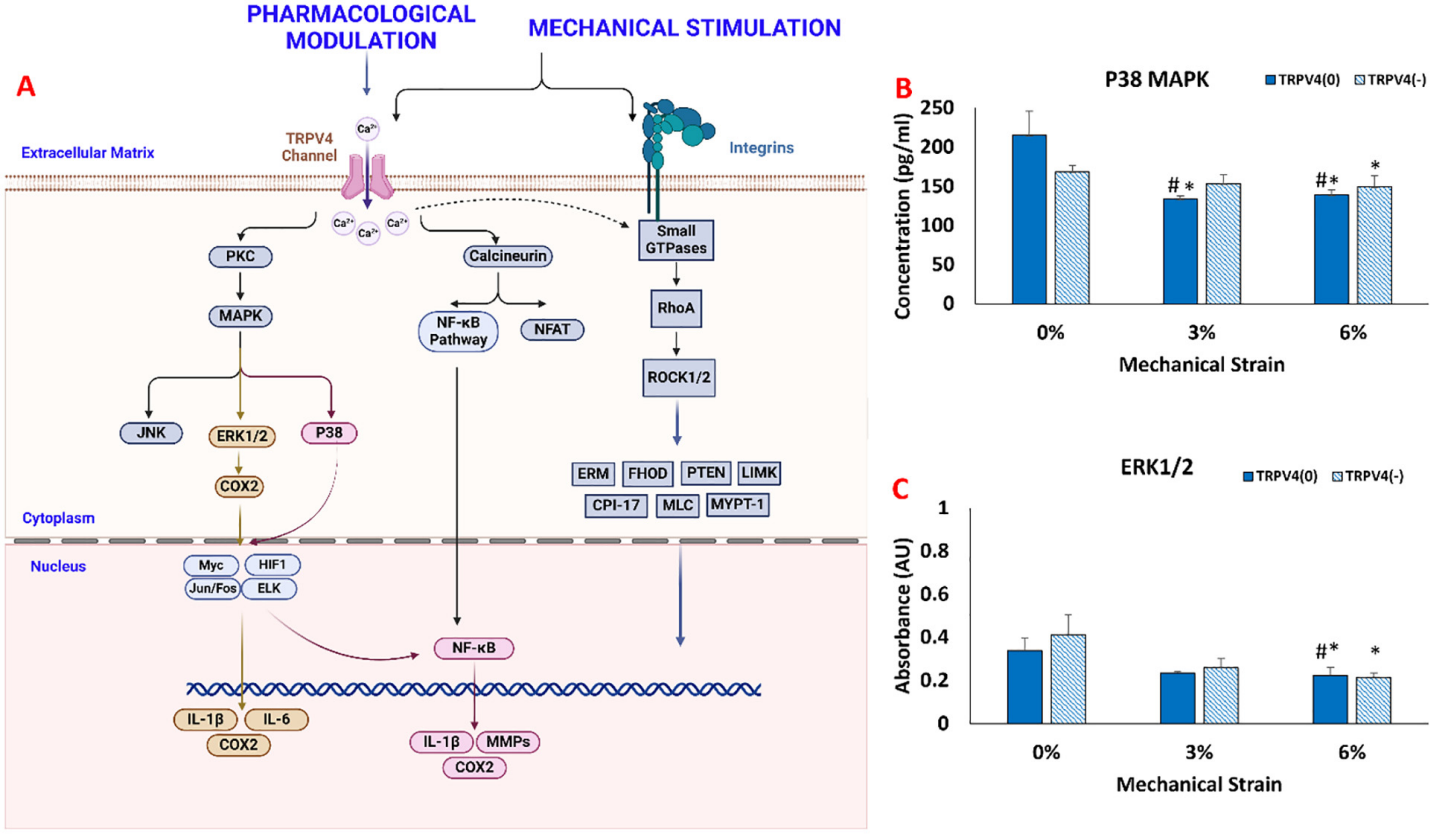
**(A)** Mechanotransduction pathway activated upon TRPV4 Inhibition and Mechanical Loading. The changes in **(B)** p38 MAPK concentration. **(C)** ERK1/ERK2 synthesis. * indicates the statistical difference between each strain group and the control group (TRPV4(0)-0%) and # indicates the statistical difference between each strain group and TRPV4(-)-0% with p<0.05.

The changes in collagen fiber density and degradation data suggested that there was a phenotypical change in macrophages upon TRPV4 inhibition and mechanical loading application. Before undertaking a thorough biological assessment, we aimed to verify whether the TRPV4 inhibitor and/or mechanical loading applied to macrophage-laden 3D tissue matrices created effects at the intra-cellular level towards immunomodulation. The mitogen-activated protein kinase (MAPK) pathway’s key components, ERK1/2, and p38 MAPKs were assessed using ELISA. As suggested in the macrophage mechanotransduction pathways (**Figure 4A**) in response to both TRPV4 inhibition and mechanical loading, the protein kinase C (PKC) is activated which further downstream to MAPK, ERK1/2, and p38 MAPKs, and triggered the inflammatory response and expression of pro-inflammatory cytokines and chemokines (21, 26).

**Figure 4B** shows that the mechanical loading and TRPV4 inhibitor affect p38 MAPK expression in different conditions. Without mechanical loading (0%), p38 MAPK expression didn’t change significantly between TRPV4(0) and TRPV4 inhibited group (TRPV4(-)). For 3% mechanical loading, p38 MAPK expression was significantly reduced to 133.53 pg/ml (p<0.05). For 6% mechanical loading, p38 MAPK expression significantly downregulated to 138.53 pg/ml for TRPV4(0) group and to 149.42 pg/ml for TRPV4(-) group.

**Figure 4C** shows the measured absorbance associated with ERK1/ERK2 protein expression in each group. Without loading (0%), ERK1/2 expression didn’t change significantly between TRPV4-inhibited (TRPPV(-)) and control groups (TRPPV(0)). Similarly, under 3% mechanical loading, the expression of ERK1/2 did not change significantly even with TRPV4 inhibition. However, under 6% mechanical loading, the expression of ERK1/2 was significantly downregulated to 0.22AU for TRPV4(0) group and to 0.21AU with TRPV4(-) group compared to counterparts in unloaded groups (0% mechanical loading).

### 3.4 Phenotypic changes in macrophages within the 3D collagen matrix upon TRPV4 inhibition and mechanical loading

#### 3.4.1 Gene expression analysis

**Figure 5:**
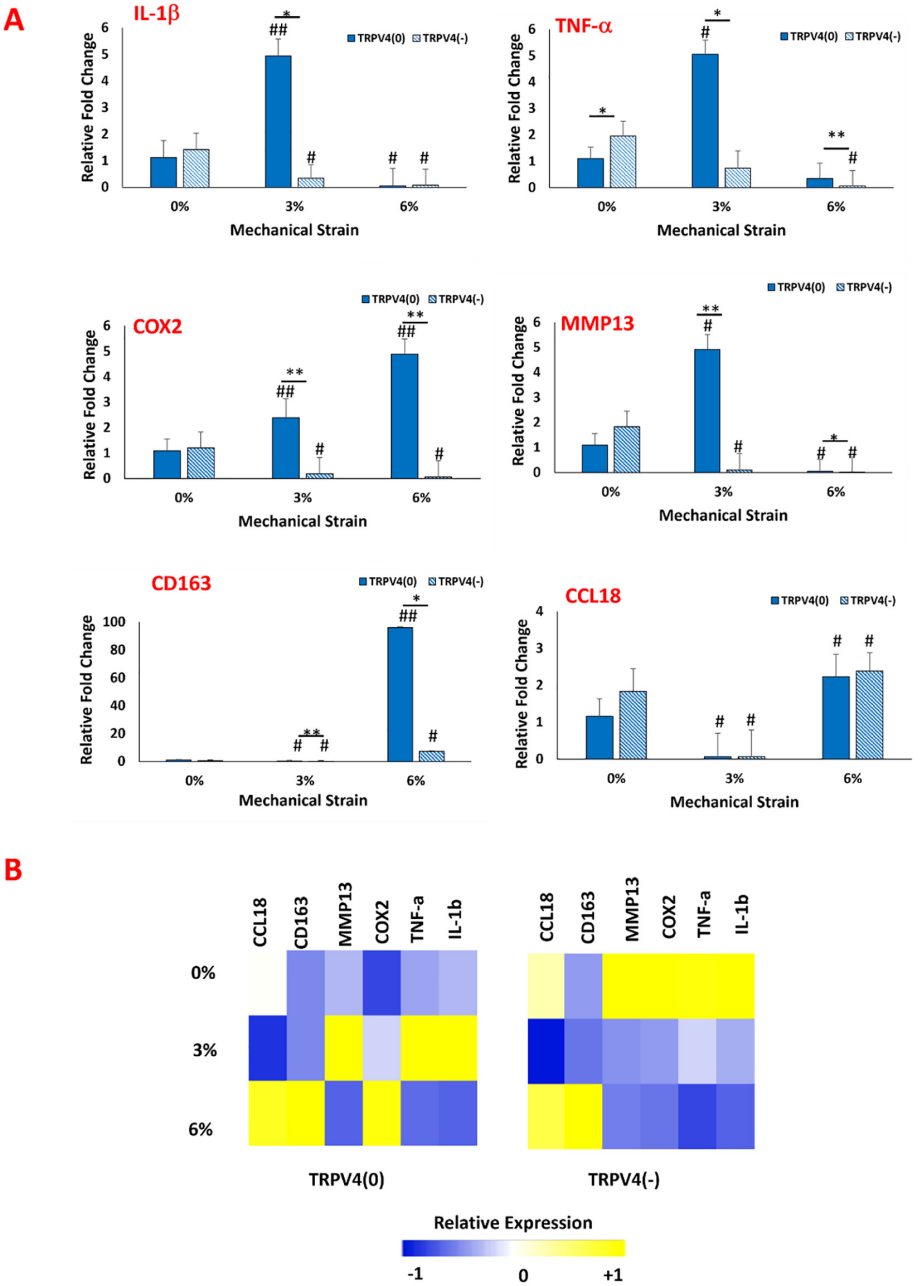
**(A)** Effect of TRPV4 inhibitor and mechanical loading on the expression of pro-and anti-inflammatory markers, and matrix degradation marker of macrophages within the 3D collagen matrix. * shows the statistical difference within the same mechanical strain group between TRPV4(0) and TRPV4(-) with a significance level of p < 0.05 and ** shows that the statistical difference has a significance level of p<0.005. # Shows the statistical difference between each experimental group and control group (TRPV4(0)-0%) with a significance level of p< 0.05 and ## shows that the statistical difference has a significance level of p<0.005. **(B)** Heatmap of pro- and anti-inflammatory gene expressions upon TRPV4 inhibitor and/or various mechanical loading application. In heatmap, yellow represents upregulation while blue represents downregulation in the gene expression.

Following the confirmation that key pathways were modulated upon TRPV4 inhibition and mechanical loading, we investigated the phenotypic changes in macrophages resulting from the modulation of the P38 and ERK1/2 pathways using gene expression analysis. The expressions of important pro-inflammatory markers (IL-1b, TNF-a, COX2), anti-inflammatory markers (CD163, CCL18), and matrix degradation markers (MMP13) were assessed. **Figure 5** shows the relative fold changes of these markers with and without TRPV4 inhibitor under various mechanical strain (0,3, and 6%) applications.

Regarding the inflammatory markers (**Figure 5A**), the IL-1β expression data suggested that TRPV4 inhibition and mechanical loading depending on mechanical strain amplitude affected its expression. For unloaded group (0% mechanical loading), IL-1β levels didn’t change significantly between TRPV4(-) and TRPV4(0) groups. Upon 3% mechanical loading, IL-1β expression increased by almost 5-fold in TRPV4(0) group compared to unloaded group. However, with the TRPV4 inhibition (TRPV4(-), IL-1β expression decreased significantly for with 3% mechanical loading group. This indicates that TRPV4 inhibition can counteract the upregulation in IL-1β expression upon 3% mechanical loading. Upon 6% mechanical loading, IL-1β expression significantly decreased in all groups regardless of TRPV4 inhibition. The TNF-α expression increased significantly by around 5-fold for 3% mechanical loaded samples compared to 0% mechanical loaded counterparts for TRPV4 (0) groups. However, with TRPV4 inhibition (TRPV4 (-)), the TNF-α expression attenuated significantly for 3% mechanical loaded samples compared to without TRPV4 inhibition at 3% mechanical loading. At 6% mechanical loading, while TNF-α expression did not significantly change for TRPV4(0) group compared to TRPV4(0)-0%, with TRPV4 inhibition, the TNF-α expression diminished significantly both compared to TRPV4(0)-0% and TRPV4(0)-6% groups. For TRPV4 (0) group, the COX2 expression increased by 2.39-fold and 4.88-fold for 3% and 6% mechanical loading groups, respectively compared to TRPV4(0)-0% group. With TRPV4 inhibition (TRPV4(-)), COX2 expression significantly decreased both 3% and 6% mechanical loading groups and both were significantly lower than their respective counterparts without inhibition (TRPV4(0)). The MMP13 gene expression data also demonstrated that for TRPV4 (0) group, upon 3% loading, the MMP13 expression increased significantly by 4.91-fold compared to TRPV4(0)-0 group. Yet, with TRPV4 inhibition (TRPV4(-)), upon 3% mechanical loading, MMP13 expression significantly decreased. Upon 6% mechanical loading, MMP13 expression further decreased without and with TRPV4 inhibition compared to 0% and 3% mechanical loaded groups with and without TRPV4 inhibition. Regarding the anti-inflammatory markers, the CD163 expression did not change significantly for 0% mechanical loading groups upon TRPV4 inhibition. Upon 3% mechanical loading, the CD163 expression decreased significantly for both TRPV4(0) and TRPV4(-) groups compared to TRPV4(0)-0% (control) group. Upon 6% mechanical loading, the CD163 expression increased significantly (p<0.005) for TRPV4(0) group almost by 100-fold compared to counterparts in 0% and 3% mechanical loading groups. Upon TRPV4 inhibition for 6% mechanical loading, CD163 expression decreased compared to without TRPV4 inhibition (TRPV4 (0)) group but still statistically significantly higher for counterparts in 0% and 3% mechanical loading groups. The CCL18 expression also did not change significantly for 0% mechanical loading groups upon TRPV4 inhibition. Upon 3% mechanical loading, there was a decrease in CCL18 expression for both TRPV4(0) and TRPV4(-) groups compared to TRPV4(0)-0% (control) group. Upon 6% mechanical loading, the CCL18 expression increased compared to counterparts in 0% and 3% mechanical loading groups for both TRPV4(0) and TRPV4(-) groups. Yet, there was no statistical difference between the expressions of CCL18 for TRPV4(0) and TRPV4(-) groups within 3% and 6% mechanical loading group.

The heatmap (**Figure 5B**) displays the overall changes in pro- and anti-inflammatory gene expressions upon TRPV4 inhibitor and/or various mechanical loading application visually. The heatmap overall demonstrates that TRPV4 inhibition significantly lowered the expression of pro-inflammatory genes upon 3% and 6% mechanical loading. Specifically, the TRPV4 inhibition along with 6% mechanical loading synergistically promoted anti-inflammatory macrophage phenotypic changes.

#### 3.4.2 Surface protein analysis

**Figure 6:**
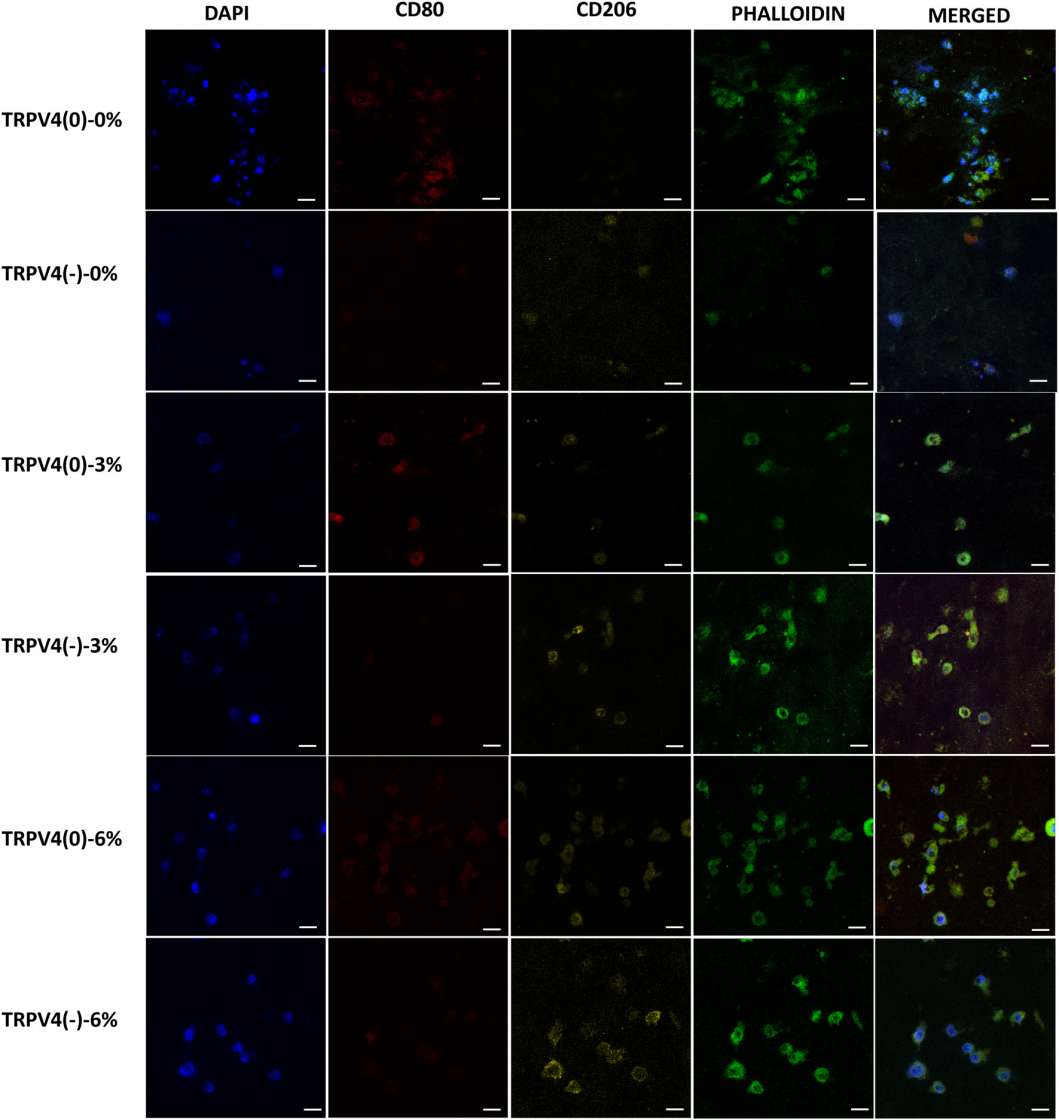
Confocal microscopy images of the cell nucleus stained with DAPI, F-actin with phalloidin, pro-inflammatory (CD80), and anti-inflammatory marker (CD206). Representative of 5 images per group (Scale bar =15 μm).

While the gene expression data may indicate phenotypic shifts towards an M1 or M2 phenotype at the mRNA level, without protein-level confirmation, it remains uncertain whether the changes in gene level translate into protein level and confirm the macrophage polarization. Immunohistochemistry was employed to visualize the binding of surface antibodies targeting CD80, a marker of pro-inflammatory macrophages, and CD206, a marker of anti-inflammatory macrophages. CD80 is associated with the pro-inflammatory response of macrophage while the CD206, also known as the mannose receptor, is a marker of alternatively activated macrophages involved in tissue repair and anti-inflammatory processes. The differential expression of these markers provides insight into the inflammatory state of the macrophages. Along with the pro- and anti-inflammatory surface antibodies, the cell nuclei and filamentous actin (F-actin) were also tagged using DAPI and phalloidin, respectively. **Figure 6** shows the confocal images of cell nuclei, pro- and anti-inflammatory markers, and F-actin of macrophages within the 3D collagen matrix exposed to various mechanical loading amplitudes with and without TRPV4 inhibitor.

The anti-inflammatory marker CD206 was upregulated upon TRPV4 inhibition, both in unloaded (0% mechanical loading) and 3% mechanical strain loading conditions. This was evident from increased CD206 expression in the TRPV4(-)-0% and TRPV4(-)-3% groups compared to their respective controls without TRPV4 inhibition. In contrast, the pro-inflammatory marker CD80 was downregulated in these conditions, indicating a shift towards an anti-inflammatory phenotype. Furthermore, when macrophages within the 3D collagen matrix were subjected to 6% mechanical loading, there was a substantial increase in CD206 expression in both TRPV4(0) and TRPV4(-) groups, highlighting the anti-inflammatory effect of 6% mechanical strain.

## 4 Discussion

Mechanical loading has been shown to regulate macrophage polarization (5) while antagonizing TRPV4 channel is used for the treatment of inflammatory disease (11–13). Yet, the effects of TRPV4 inhibition and mechanical strain amplitude on macrophage phenotype and ECM remodeling remain elusive. This study for the first time investigated how pharmacological inhibition of TRPV4 combined with mechanical strain influences the polarization and function of M1 macrophages within 3D collagen matrices, assessing the expression and synthesis of pro- and anti-inflammatory markers and matrix remodeling to understand the interplay between mechanical and pharmacological modulation in macrophage phenotype commitment.

It should be also noted that the current study is also innovative for studying the effect mechanical loading and TRPV4 inhibitor on macrophage polarization within the 3D collagen matrix (**Figure 1**). The mechanoresponsiveness of macrophages has traditionally been studied using two-dimensional (2D) cell culture plates, such as petri dishes and flasks, or *in vivo* animal models. While 2D *in vitro* studies are cost-effective and utilize straightforward protocols, macrophages on flat surfaces exhibit altered cellular functions and polarization potential compared to those embedded within three-dimensional (3D) matrices (27). This discrepancy underscores the importance of 3D *in vitro* studies, which offer a more physiologically relevant environment that better mimics the extracellular matrix conditions encountered by macrophages *in vivo*. Unlike studies focusing on macrophage mechanotransduction, which often use 2D cultures or animal models, TRPV4 research frequently relies on *in vivo* models to gain more physiologically relevant data (9, 28). However, systemic modulation of TRPV4 channels in these models can lead to off-target effects due to the widespread presence of TRPV4 across various mammalian cell membranes. Additionally, *in vivo* models make it challenging to isolate the specific effects of TRPV4 on macrophage phenotypic commitment. Thus, 3D *in vitro* models present a valuable alternative for 2D *in vitro* and *in vivo* studies.

Following synthesizing pro-inflammatory macrophage encapsulated within 3D collagen matrices, comprehensive structural and molecular analyses were conducted. First, the anti-TRPV4 antibodies were visualized upon various mechanical loading (0,3,6%) with and without TRPV4 inhibitor. The immunofluorescence image (**Figure 2A**) and image analysis (**Figure 2B**) demonstrated that TRPV4 expression progressively increased with mechanical loading in the absence of a TRPV4 inhibitor, indicating a strain-dependent activation of the channel (**Figure 2**). This suggests that mechanical strain can enhance TRPV4 activity, possibly influencing macrophage behavior. Conversely, the presence of a TRPV4 inhibitor reduced TRPV4 expression, particularly at higher strain levels (3% and 6%), highlighting the effectiveness of TRPV4 inhibition in controlling channel activation. This finding is consistent with the literature. For instance, Xu et al. (29) demonstrated that increasing cyclic stretch time up to 24 hours increased the TRPV4 expression gradually and can be significantly reduced by TRPV4 inhibitor treatment (29). This differential regulation of TRPV4 expression underlines the channel’s responsiveness to mechanical cues and pharmacological intervention, setting the stage for exploring its role in macrophage mechanotransduction.

Building on the modulation of TRPV4 expression, this study examined how these changes impact the structural integrity of the extracellular matrix. The structural assessment using Masson’s trichrome and B-CHP staining revealed distinct differences in collagen fiber density and degradation upon mechanical loading and TRPV4 inhibition (**Figure 3**). Masson’s trichrome staining showed increased collagen density in the group with combined TRPV4 inhibition and 6% mechanical loading, suggesting reduced or halted collagen degradation, potentially through the modulation of macrophage phenotype. This observation aligns with the findings of Seyeon Oh et al. (30), who reported that macrophage polarization toward an anti-inflammatory phenotype increased collagen fiber density in a mouse model. These results may indicate that TRPV4 inhibition combined with mechanical loading can enhance matrix accumulation and the functional properties of the extracellular matrix. The TRPV4(0)-3% group exhibited the most significant collagen degradation, emphasizing the role of TRPV4 in matrix remodeling under mechanical strain. This can be attributed to increased inflammation and elevated enzymatic activity of matrix metalloproteinases (MMPs), which directly increase collagen degradation and can synergize to further accelerate matrix breakdown. In contrast, TRPV4 inhibition mitigated these effects, resulting in thicker collagen fibers and suggesting that blocking TRPV4 can protect the extracellular matrix from mechanical strain-induced degradation. These findings underscore the crucial role of TRPV4 in regulating collagen integrity through macrophage-mediated mechanisms, highlighting the interplay between mechanical strain, TRPV4 activity, and matrix remodeling.

Further, we investigated how TRPV4 and mechanical loading affect macrophage signaling pathways, focusing on the MAPK pathway, specifically p38 and ERK1/2 (**Figure 4**). The MAPK pathway was chosen because of its involvement in response to mechanical stress and calcium influx activation mechanisms (31–33). For instance, Jenny Papakrivopoulou et al. (34) found that excessive cyclic cardiac loading activated MAP kinase family members, including ERK and p38 MAPK, in rat cardiac fibroblasts. In this study, the expression of p38 MAPK and ERK1/2 was differentially modulated by TRPV4 inhibition and mechanical strain. Specifically, the mechanical loading without TRPV4 inhibition significantly downregulated p38 MAPK and ERK1/2, especially under 6% strain, suggesting that high mechanical loading might override the TRPV4 channel’s influence on these pathways. **Figure 4** suggesting that the greatest anti-inflammatory shift in M1-laden macrophages was linked with the lowest MAPK activity, consistent with previous studies demonstrating that MAPK suppression inhibits inflammation *in vivo* (35, 36) and *in vitro* (37).

The effect of TRPV4 inhibitor and mechanical loading-induced MAPK suppression further studied using pro-and anti-inflammatory gene expression analysis (**Figure 5**). The significant upregulation of pro-inflammatory markers such as IL-1β and TNF-α under 3% strain without TRPV4 inhibition underscores the channel’s involvement in promoting an inflammatory phenotype. Enhanced expression of anti-inflammatory markers like CD163 and CCL18 under 6% strain, particularly in TRPV4-inhibited groups, further supports the role of TRPV4 blockade in promoting a reparative macrophage phenotype. This shift towards an anti-inflammatory state is particularly significant in the context of extracellular matrix (ECM) integrity, as increased levels of inflammatory cytokines like TNF-α and IL-1β have been correlated with collagen degradation in tissues (38). M1-laden macrophages exposed to 3% mechanical strain exhibited the highest levels of TNF-α, IL-1β expressions (**Figure 5**), and collagen degradation (**Figures 3A, B**). Matrix metalloproteinases (MMPs), upregulated in response to pro-inflammatory cytokines, also play a crucial role in collagen degradation (39, 40). The gene expression data (**Figure 5**) revealed that TRPV4 inhibition attenuates MMP13 expression, which corresponds with higher collagen content subjected to mechanical loading (**Figure 3A** and 3B). The combined influence of mechanical strain and TRPV4 inhibition on gene expression profiles suggests that mechanical loading exerts a more pronounced effect on the upregulation of inflammatory genes like IL-1β compared to TRPV4 inhibition alone. However, the attenuation of TNF-α expression was most significant when TRPV4 inhibition combined with 6% mechanical strain. Overall gene expression data demonstrated in heatmap (**Figure 5B**) demonstrated that the most substantial attenuation of inflammatory gene expression occurs when M1 macrophages experience concurrent TRPV4 inhibition and mechanical loading. The reduced expressions of inflammatory markers such as IL-1β, TNF-α, COX2, and MMP13 in TRPV4-inhibited groups underlines the channel’s role in mediating the cellular response to mechanical stimuli, providing valuable insights into the development of anti-inflammatory strategies in mechanically dynamic environments.

To confirm the observed phenotypic shifts at the transcriptional level, the macrophage surface proteins associated with polarization states were tagged and visualized. Surface protein analysis using confocal microscopy agreed with the gene expression findings, demonstrating that TRPV4 inhibition increased the expression of CD206, an anti-inflammatory macrophage marker, while decreasing CD80, a pro-inflammatory marker (**Figure 6**). The pronounced upregulation of CD206 under 6% strain with TRPV4 inhibition strongly indicates that mechanical strain, when combined with TRPV4 inhibition, can effectively drive macrophages towards an M2-like anti-inflammatory phenotype. These surface marker changes at the protein level validate the transcriptional shifts observed, confirming that TRPV4 modulation and mechanical loading significantly impact macrophage polarization.

## 5 Conclusion

This study provides novel insights into the complex interplay between TRPV4 inhibition and mechanical loading on macrophage polarization and ECM remodeling within 3D collagen matrices. It was demonstrated that mechanical loading, especially when combined with TRPV4 inhibition, significantly modulates macrophage phenotypes and influences collagen integrity, highlighting the critical role of TRPV4 in mechanotransduction and inflammation. TRPV4 inhibition reduced pro-inflammatory signaling and MMP activity, which are known contributors to ECM degradation, while promoting anti-inflammatory macrophage polarization, particularly under higher mechanical strain (6%).The study also underscores the importance of using 3D *in vitro* models, as they provide a more physiologically relevant environment that closely mimics *in vivo* conditions compared to traditional 2D cultures. This approach effectively isolates the specific effects of TRPV4 on macrophage behavior, overcoming limitations associated with systemic modulation in animal models. The TRPV4 inhibition combined with mechanical loading not only shifts macrophages towards a reparative phenotype but also preserves ECM integrity by attenuating inflammatory signaling pathways. These results have important implications for developing targeted anti-inflammatory therapies in mechanically dynamic tissues and diseases where inflammation and matrix degradation are critical concerns.

## Data Availability

The datasets presented in this study can be found in online repositories. The names of the repository/repositories and accession number(s) can be found in the article/supplementary material.
